# Embodied Cross-Modal Interactions Based on an Altercentric Reference Frame

**DOI:** 10.3390/brainsci14040314

**Published:** 2024-03-27

**Authors:** Guanchen Guo, Nanbo Wang, Chu Sun, Haiyan Geng

**Affiliations:** 1 School of Psychological and Cognitive Sciences, Beijing Key Laboratory of Behavior and Mental Health, Peking University, Beijing 100871, China; guoguanchen@pku.edu.cn (G.G.); sunchu@pku.edu.cn (C.S.); 2 Department of Psychology, School of Health, Fujian Medical University, Fuzhou 350122, China; wangnb@fjmu.edu.cn

**Keywords:** visual perspective-taking, cross-modal interactions, embodied processing, altercentric reference frame

## Abstract

Accurate comprehension of others’ thoughts and intentions is crucial for smooth social interactions, wherein understanding their perceptual experiences serves as a fundamental basis for this high-level social cognition. However, previous research has predominantly focused on the visual modality when investigating perceptual processing from others’ perspectives, leaving the exploration of multisensory inputs during this process largely unexplored. By incorporating auditory stimuli into visual perspective-taking (VPT) tasks, we have designed a novel experimental paradigm in which the spatial correspondence between visual and auditory stimuli was limited to the altercentric rather than the egocentric reference frame. Overall, we found that when individuals engaged in explicit or implicit VPT to process visual stimuli from an avatar’s viewpoint, the concomitantly presented auditory stimuli were also processed within this avatar-centered reference frame, revealing altercentric cross-modal interactions.

## 1. Introduction

People possess a multitude of beliefs, opinions, and ideas stemming from their diverse perceptual experiences. Given this cognitive diversity, a crucial aspect of fostering effective social interactions lies in the ability to comprehend others’ visual experiences [1,2,3]. To achieve this, individuals typically imagine themselves standing in others’ positions to process their surrounding visual world [4,5,6,7]. For instance, ground workers need to adopt the visual perspective of the high-altitude manipulator and provide precise instructions for positioning the equipment accurately. Therefore, envisioning others’ visual experience plays a critical role in social interactions [8]. However, in our daily lives, apart from processing visual information in isolation, we consistently encounter inputs from diverse modalities. How individuals process and integrate these sensory inputs while adopting others’ perspectives remains an intriguing question. Investigating these inquiries could enhance our comprehension of how information from multiple modalities is harnessed to construct a cohesive experience that facilitates individuals in inferring another individual’s perceptual world.

In self-perspective processing, sensory information from multiple modalities can interact with each other to construct a coherent and comprehensive understanding of the surrounding environment [9,10,11,12]. This phenomenon, known as cross-modal interactions, is robust and widespread in individuals’ egocentric processing. A fundamental prerequisite for such interactions is the spatial correspondence across different modalities [13,14]. For instance, participants exhibited improved accuracies in localizing a visual target when it was presented on the same side as an auditory stimulus compared to when it was presented on the opposite side [13,15]. Additionally, participants demonstrated enhanced accuracies in identifying motion direction when visual motion was accompanied by congruent auditory motion compared to incongruent motion [16,17]. Moreover, participants exhibited improved speed estimation [18] and trajectory tracking performance [19,20] in audiovisual congruent situations.

However, existing studies regarding perceptual processing in an altercentric reference frame have primarily focused on a single visual modality, wherein individuals mentally visualize how one or more objects appear from another person’s viewpoint (visual perspective-taking, VPT [5,21,22,23,24]). Typically, participants were instructed to adopt another person’s (or a human avatar’s) visual perspective to observe one or more visual targets and then respond to their contents or judge their relative spatial locations from the adopted perspective. Many researchers proposed that individuals perceived and predicted others’ visual experiences in an embodied manner [25,26,27,28]. That is, individuals engaged in visual processing as if they were physically present at another person’s location after conducting a mental-body transformation [5]. In this way, people would “stand in others’ shoes”, “see through others’ eyes”, and obtain an immersive visual experience. For instance, when judging whether a letter R lying flat on the table was upright or mirrored, participants reacted faster when the angular discrepancy between the letter and participants’ line of sight decreased. More importantly, when another person was sitting beside the table, the decrease in the angular discrepancy between the letter and that person’s line of sight produced the same effect [7,29,30]. Also, our previous study showed that when participants were adopting an avatar’s visual perspective to perceive moving dots, a corresponding embodied motion adaptation had been induced, which resembled motion adaptation evoked from one’s self-perspective [6]. Meanwhile, the left-neglect symptoms caused by right-hemisphere lesions were compensated when the patients adopted the visual perspective of another person sitting opposite to them [31]. All these results suggest that people complete VPT by transforming themselves into the adopted person’s position and then performing an embodied visual processing in an altercentric reference frame.

Based on the above evidence, the embodied processing mechanism in VPT encourages us to anticipate the possibility of altercentric cross-modal interactions. When people mentally transform their bodies into the adopted person’s location, the reference frame they use should change from an egocentric to an altercentric one. Given the embodied nature of VPT, altercentric processing can be viewed as a form of virtual-egocentric processing. In this scenario, the concomitantly presented non-visual (e.g., auditory) information should also be processed in that altercentric reference frame, as if it was perceived by people’s “re-located head” after the mental-body transformation. Therefore, it is likely that both the visual and the auditory processing during VPT occur in this virtual-egocentric reference frame, and that the information from these two modalities can interact in an altercentric manner along with the embodied process.

Specifically, this idea is in line with the transformation-uniform theory proposed in the field of egocentric cross-modal interactions [32]. This view regards cross-modal interactions as a process of coordinating information from different modalities to unify them in a common reference frame [33,34,35]. For example, while visual stimuli are commonly perceived within the eye-centered reference frame, auditory stimuli are typically interpreted within the head-centered reference frame [36,37,38]. Individuals possess the ability to transform auditory stimuli from the head-centered to the eye-centered reference frame to assess whether or not the locations of the stimuli from the two modalities correspond [36,37]. Conversely, individuals can also convert visual information into the head-centered reference frame for comparable purposes [39,40]. This unification process determines whether the information from two modalities can interact or not. Based on the transformation-uniform view, we can reasonably expect the occurrence of altercentric cross-modal interactions in that people may rely on embodied processing to unify visual and auditory information into the altercentric reference frame.

To verify the existence of altercentric cross-modal interactions, an experimental paradigm is needed to exclude potential confusions that come from egocentric interactions. For example, visual and auditory stimuli should be designed to correspond in space only in the altercentric reference frame, but not in the egocentric reference frame. Therefore, we designed a visual scene in which an avatar was located on the left or the right side of the screen, facing the visual stimuli presented in the center of the screen. At the same time, an auditory stimulus was presented to the participants through headsets. Specifically, the visual stimuli were presented in the up-down dimension from the participant’s visual perspective and would be perceived as being presented in the left-right dimension from the avatar’s visual perspective (in the avatar-centered reference frame). Relatively, the auditory stimuli were presented in a head-centered reference frame [41], and the acoustic source would be located in the left-right dimension based on the participants’ heads [37]. Given the proprioception attribute of the auditory information received by the headset [42], if participants mentally transform their bodies into the avatar’s location during VPT, they should feel like hearing an auditory stimulus emanating from either the left or the right side of their “re-located head” (in the avatar-centered reference frame). Correspondingly, visual and auditory stimuli would both be processed in an embodied manner in the avatar-centered reference frame, and their spatial correspondence in the left-right dimension would lead to a cross-modal interaction. However, if embodied processing did not occur, both visual and auditory inputs would be processed in the participant’s egocentric reference frame. Cross-modal interactions would be impossible because the visual stimuli (in the up-down dimension) and the auditory stimuli (in the left-right dimension) are spatially mismatched with each other.

In total, we conducted six experiments to investigate the existence of altercentric audiovisual interactions. Experiment 1a demonstrated that auditory stimuli could be processed and interact with visual stimuli in the altercentric reference frame during a VPT task, as evidenced by the altercentric auditory cueing effect on visual targets in a location identification task. Experiment 1b further elucidated that this cueing effect cannot be attributed to an alternative explanation of auditory bias by removing the avatar and implementing an equivalent response rule. Experiment 2 re-examined the existence of altercentric cross-modal interactions in motion processing and revealed that auditory motion improved participants’ ability to discriminate the direction of the simultaneously presented visual motion within the altercentric reference frame. In Experiment 3a, we demonstrated the persistence of altercentric cross-modal interactions even when the avatar was rendered as task-irrelevant, thereby suggesting participants’ inherent inclination to spontaneously and implicitly adopt an altercentric reference frame. Finally, Experiments 3b and 3c observed the disappearance of the cross-modal interactions when replacing the human avatar with an inanimate object or reorienting the avatar away from the visual stimuli, respectively. The results validated that the observed outcomes in Experiment 3a were specifically attributed to the implicit adoption of the human avatar’s visual perspective and embodied processing from its viewpoint, rather than being influenced by other confounding factors. Collectively, these findings provide compelling evidence supporting the existence of altercentric audiovisual interactions when adopting others’ visual perspectives.

## 2. Experiment 1a

In Experiment 1a, we investigated whether both the visual targets and the additional auditory stimuli could be processed in an embodied manner in the altercentric reference frame when people were explicitly performing a VPT task. As mentioned above, we combined a classic VPT task with the multisensory processing paradigm. An auditory cue was presented to participants’ left or right ears via a headset, and participants were asked to judge the location (left or right) of a subsequent visual target from an avatar’s visual perspective. Specifically, the visual target was presented in the up-down dimension from the participant’s viewpoint but in the left-right dimension from the avatar’s visual perspective. Therefore, if participants mentally transformed their bodies into the avatar’s location in an embodied manner, the auditory cue and the visual target could correspond spatially in the avatar-centered reference frame and the auditory cues were expected to elicit a cross-modal cueing effect on participants’ location discrimination of visual targets. Otherwise, if embodied processing did not occur, the visual targets and the auditory cues would be processed in the participant’s egocentric reference frame. Correspondingly, their spatial locations were orthogonal to each other in the egocentric reference frame: The visual targets were in the up-down dimension while the auditory cues were in the left-right dimension. As a result, the cueing effect should not occur.

### 2.1. Method

#### 2.1.1. Participants

The sample size of Experiment 1a was determined through a priori power analysis using G*Power 3.1 [43]. After reviewing studies employing similar paradigms that reported effect sizes (Cohen’s *d*) of 0.93 for position discrimination tasks in VPT [5], as well as 1.06 and 1.54 for cueing tasks and the cross-modal form [44,45], we set an empirically large effect size (Cohen’s *d*) of 0.80. With this effect size, a power of 0.90, and an alpha level of 0.05, the power analysis conducted with a paired sample *t*-test yielded an estimated sample size of 19.

Based on our experience employing this paradigm, we recruited a larger number of participants than the desired quantity to account for potential exclusions, such as incomplete task performance or a low hit rate for catch trials. Twenty-four participants from Peking University were recruited for Experiment 1a. All participants reported normal or corrected-to-normal vision and were checked to confirm their normal hearing. After excluding four participants due to their low hit rate in catch trials (<80%), the remaining 20 participants (13 females, *M* = 22.5, *SD* = 3.5) were included in the data analyses (their hit rate was 93.3% ± 5.0% [*M* ± *SD*]).

#### 2.1.2. Apparatus and Stimuli

The experiment was conducted in a dimly lit, quiet room. Participants’ eyes were 50 cm away from a 27-inch LCD BenQ computer monitor (Resolution: 1920 × 1080; Refresh rate: 100 Hz) and a chin rest was used to maintain their head position. The experiments were programmed and run in MATLAB 2018, using Psychtoolbox-3. Data were analyzed using JASP 0.16.4.0.

**Figure 1:**
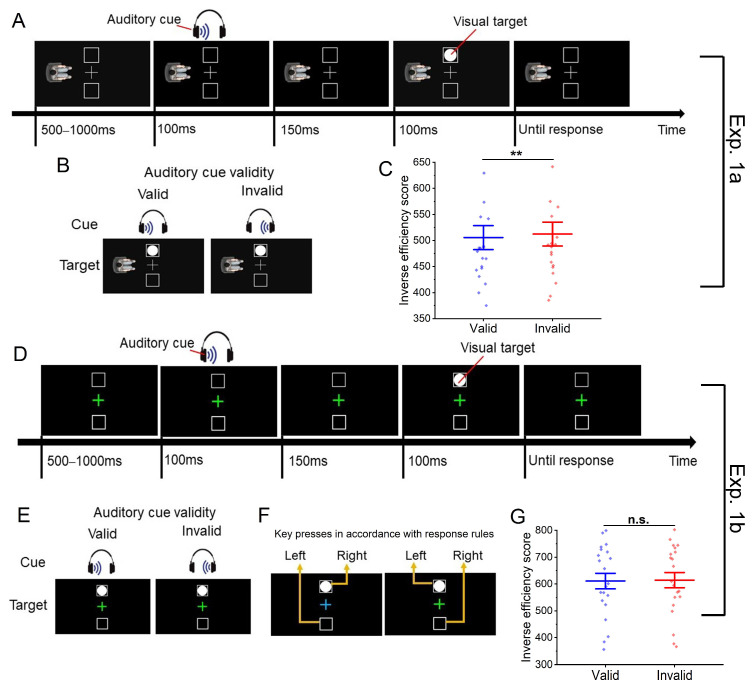
(**A**,**D**) Experimental procedures. An exemplary trial in the valid condition featuring a “left” auditory cue and a “left” visual target in Experiment 1a (**A**). In Experiment 1b (**D**), the color of the fixation cross serves as an indicator of response rules, while the avatar is not presented. (**B**,**E**) Experimental conditions. A demonstration of the valid and invalid conditions corresponding to a “left” visual target, from a left avatar’s visual perspective (**B**), or according to response rules (**E**). (**F**) Response rules in Experiment 1b. The mapping between colors and response rules is counterbalanced across participants. (**C**,**G**) Experimental results. Participants’ inverse efficiency scores in the two conditions in Experiments 1a (**C**) and 1b (**G**). Error bars indicate one standard error. ** *p* < 0.01. “n.s.” indicates the lack of statistical significance.

The visual stimuli (see Figure 1A) were presented against a black background (RGB: 0, 0, 0). A white fixation cross (1.6° × 1.6°) was displayed at the center of the screen throughout each trial, and two white squares (0.8° × 0.8°) were located above and below the fixation cross, respectively, with their centers 3.2° away from the center of the screen. The visual target was a white circle presented in one of the squares. A human avatar was located either on the left or the right side of the screen, facing the center of the screen. The avatar was created using 3dsMax 2021 and was presented 7° away from the center of the screen.

The auditory stimuli were a 1000 Hz sine tone with a duration of 100 ms, including 10 ms cosine ramped rise and fall envelops. There are three types of sounds (“left”, “right”, and “both”) delivered through headsets (AKG K77). In the “left” and the “right” situations, auditory stimuli were presented only on the corresponding side of the headset. In the “both” situation, stimuli were presented on both sides of the headset. At the beginning of the experiment, each participant completed a pure auditory block to familiarize themselves with the auditory stimuli and check their hearing. During this block, participants could adjust the loudness of the auditory stimuli to a comfortable level (about 60–75 dB sound pressure level).

#### 2.1.3. Design and Procedure

We employed a one-factor (auditory cue validity: valid vs. invalid) within-participant design in Experiment 1a. When the acoustic source of the auditory cue presented from the headset was consistent with the location of the visual target in the avatar-centered reference frame, this auditory cue was considered valid (e.g., an auditory cue presented in the left side of the headset was valid when the visual target was presented on the left side of the fixation cross from the avatar’s perspective, as shown in Figure 1B). Otherwise, it was invalid. Additionally, the auditory cue was designed with a validity proportion of 50% and without explicit instruction to attend to it, in order to investigate its automatic attentional capture (as evidenced by the cueing effect).

Each formal trial began with a fixation cross presented at the center of the screen, with two squares displayed above and below the fixation and an avatar located on the left or the right side of the fixation (see Figure 1A). After a random duration of 500–1000 ms, an auditory cue was presented to participants’ left or right ears via the headset for 100 ms. Then, following a 150 ms delay, a visual target appeared in one of the squares for 100 ms (here, the stimulus onset synchrony (SOA) between the cue and the target was set to be 250 ms, through which the cue could produce a facilitating effect on the identification of the target [44]). Participants were instructed to adopt the avatar’s visual perspective to respond to the location of the targets by pressing the “z” or “x” key (indicating left or right, respectively). They were required to respond as quickly and accurately as possible. Catch trials were included in the experiment to prevent participants from ignoring the auditory information, in which the sound was presented in both sides of the headset, and participants were instructed to press the digital “0” key and need not respond to the visual target if they heard the “both” stimuli.

Each participant completed 5 blocks of trials. Each block consisted of 100 trials, including 96 formal trials and 4 catch trials, leading to a total of 20 catch trials and 480 formal trials (240 trials in each condition). The positions of the avatar, the target, and the auditory cue were randomized within each block. Participants additionally underwent a practice block of 32 trials (including 4 catch trials) at the beginning of the experiment to familiarize themselves with the experimental procedure. They received feedback at the end of each trial in the practice block, but not in the formal blocks.

### 2.2. Results

Trials with reaction times (RTs) between 250 ms to 1000 ms were included in the data analyses (96.6% of total trials). Considering the presence of the speed-accuracy trade-off, we employed the inverse efficiency score as the dependent variable to assess the efficiency of participants’ responses. This score was calculated by dividing the average RT in trials with correct responses by the proportion of accurate-response trials within each condition [46,47]. A paired sample *t*-test was conducted on this index and revealed a significant influence of auditory cue validity on participants’ efficiency in target discrimination, *t*(19) = 3.33, *p* = 0.003, Cohen’s *d* = 0.75. Participants were more efficient (took less time to make a correct response) when the cue was valid (*M* = 505.7, *SE* = 23.1) compared to when it was invalid (*M* = 512.3, *SE* = 22.9; see Figure 1C).

### 2.3. Discussion

The significant differences in inverse efficiency scores between the valid and invalid conditions indicated that individuals possess the ability to automatically process auditory cues in an embodied manner. Specifically, participants exhibited better performance in localizing visual targets from the avatar’s visual perspective when the proceeding auditory cue was valid compared to when it was invalid (cross-modal cueing effect [13,15]). Since the validity of the auditory cue relied on encoding within the avatar-centered reference frame, these results suggest that individuals processed auditory stimuli based on an altercentric reference frame when explicitly adopting the avatar’s perspective for visual stimuli processing.

## 3. Experiment 1b

The purpose of Experiment 1b was to investigate an alternative explanation for the findings in Experiment 1a. Specifically, it was hypothesized that participants might not adopt the perspective of the avatar but rather interpret it as an indicator for applying response rules. In this regard, when the avatar appeared on the left side of the screen, participants could associate up and down targets with left and right key presses, respectively, and vice versa when the avatar appeared on the right side. Consequently, in conditions where the instructed key presses and auditory stimuli were spatially congruent (the valid condition), participants exhibited better performance compared to conditions where these two locations differed (the invalid condition).

To explore this explanation, we removed the avatar and instead employed color changes in the fixation as indicators for response rules. Specifically, participants were instructed to respond to up and down targets with left and right key presses, respectively, or vice versa depending on the color of the fixation. We anticipated that if this alternative explanation held true, a cueing effect would still exist. Otherwise, it would suggest that participants did adopt the avatar’s perspective rather than considering it solely as a rule indicator.

### 3.1. Method

#### 3.1.1. Participants

With a similar experimental design, Experiment 1b utilized the identical estimated number of participants (19 individuals) as Experiment 1a. Twenty-five participants from Peking University were recruited for Experiment 1b. All participants reported normal or corrected-to-normal vision and were checked to confirm their normal hearing. After excluding four participants due to their low hit rate in catch trials (<80%), the remaining 21 participants (7 females, *M* = 19.3, *SD* = 1.7) were included in the data analyses (their hit rate was 92.1% ± 7.0% [*M* ± *SD*]).

#### 3.1.2. Apparatus and Stimuli

The apparatus and the stimuli were identical to those in Experiment 1a, with the exception that the avatar was not presented, and the fixation cross could be either blue (RGB: 0, 0, 255) or green (RGB: 0, 255, 0).

#### 3.1.3. Design and Procedure

The design and procedure of this experiment closely resembled those employed in Experiment 1a, with the exception that the avatar was excluded, and the fixation cross underwent a random color alteration, either in blue or green in each trial (see Figure 1D). Participants were instructed to respond to visual targets appearing above or below by pressing the “z” or “x” key, respectively, when the fixation was presented in one color (e.g., green, see Figure 1F) and by pressing the “x” or “z” key when the fixation appeared in the other color (e.g., blue). The mapping between colors and response rules was counterbalanced across participants. A trial where auditory stimuli occurred at positions corresponding to the required key presses according to the rules was considered valid (see Figure 1E). Otherwise, it was invalid.

### 3.2. Results

Trials with reaction times (RTs) between 250 ms to 1000 ms were included in the data analyses (89.8% of total trials). We conducted a paired sample *t*-test to examine the impact of auditory cue validity (valid vs. invalid) on participants’ inverse efficiency scores. The difference in inverse efficiency scores between the valid condition (*M* = 611.1, *SE* = 29.0) and the invalid condition (*M* = 614.7, *SE* = 28.3) was not significant, *t*(20) = 0.94, *p* = 0.358, Cohen’s *d* = 0.21 (see Figure 1G).

### 3.3. Discussion

The results of Experiment 1b revealed no significant difference in participants’ performance between the valid and the invalid conditions, thereby refuting the possibility that participants were guided by a specific rule for responding. Thus, the alternative explanation for our findings in Experiment 1a was excluded.

Subsequently, we intended to further validate the existence and generalizability of this altercentric cross-modal interaction in motion perception scenarios. Motion processing demands sustained attention and cognitive engagement [48], enabling individuals to envision how stimuli are perceived through others’ eyes for a period of time [6]. In other words, the temporal span of embodied processing is also extended. Therefore, would concurrently present non-visual stimuli also undergo altercentric processing and elicit cross-modal interactions during such prolonged embodied perception situations? Exploring this phenomenon will contribute to a more comprehensive understanding of altercentric cross-modal interactions.

## 4. Experiment 2

In Experiment 2, we aimed to further validate the existence of altercentric cross-modal interactions in motion perception scenarios. Specifically, participants were instructed to adopt an avatar’s visual perspective to discriminate the direction of visual motion while simultaneously being presented with an auditory motion stimulus through headsets. The perceived direction of the visual motion aligned with the up-down dimension from the participant’s visual perspective but corresponded to the left-right dimension from the avatar’s perspective, while the perceived direction of the auditory motion was based on a head-centered reference frame and corresponded to the left-right dimension. Therefore, spatial congruency of motion stimuli from two modalities was contingent upon participants’ adoption of the avatar-centered reference frame. We hypothesized that embodied processing within this frame would result in enhanced performance in identifying visual motion direction when paired with a congruent-direction auditory motion compared to an incongruent one. In contrast, if processed within the participants’ egocentric reference frame, visual and auditory motion would be spatially unrelated to each other and no improvement in visual discrimination would be observed.

### 4.1. Method

#### 4.1.1. Participants

The sample size of Experiment 2 was determined through a priori analysis using G*Power 3.1 [43]. Upon reviewing prior studies on audiovisual integration, which reported effect sizes (Cohen’s *d*) ranging from 0.81 to 3.30 for audiovisual congruency [17,49,50], we estimated an empirically large Cohen’s *d* of 0.80. With this effect size, a power of 0.90, and an alpha level of 0.05, the power analysis conducted with a paired sample *t*-test yielded an estimated sample size of 19.

Twenty-one participants from Peking University were recruited for Experiment 2. All participants reported normal or corrected-to-normal vision and were checked to confirm their normal hearing. After excluding one participant whose hit rate in catch trials was low (<80%), the remaining 20 participants (12 females, *M* = 21.9, *SD* = 2.1) were included in the data analyses (their hit rate was 95.5% ± 5.0% [*M* ± *SD*]).

#### 4.1.2. Apparatus and Stimuli

The apparatus used in Experiment 2 was the same as that in Experiment 1a. The visual background, fixation cross, and human avatar were also unchanged.

**Figure 2:**
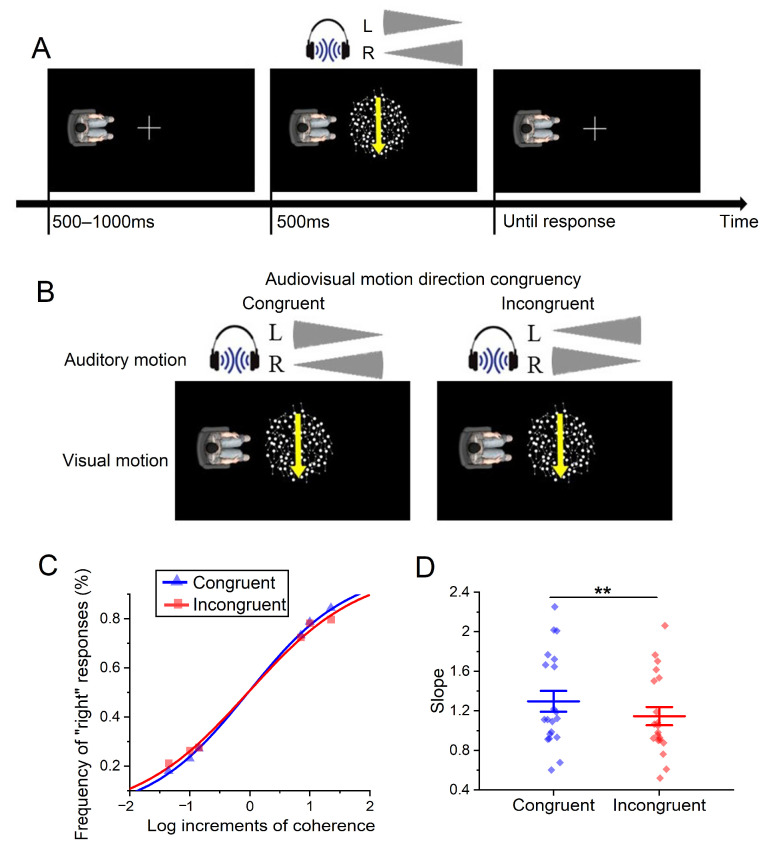
(**A**) Experimental procedure. An exemplary trial in the congruent condition with a “rightward” auditory motion and a “rightward” visual motion. The yellow arrow indicating the direction of coherent moving dots is not presented during the experiment. The grey fan-shaped symbols represent loudness changes in the left (L) and the right (R) channels of the headset. (**B**) Experimental conditions. A demonstration of the congruent and incongruent conditions corresponding to a “rightward” visual stimulus from a left avatar’s visual perspective. (**C**,**D**) Experimental results. The psychophysical curve (**C**) illustrates the group-level frequency of reporting “rightward” at different coherence levels of visual motion, where positive values indicate rightward motion and negative values indicate leftward motion. The slopes of the psychophysical curves (**D**) in the two conditions. Error bars indicate one standard error. ** *p* < 0.01.

The visual targets were a group of moving dots located at the center of the screen. Each dot was 0.2° and the aggregated group had a diameter of 12°. The group contained 500 dots that moved at a speed of 5°/s for 500 ms (see Figure 2A). Some of the dots moved coherently in a specific direction, that was, either from up to down or from down to up from the participant’s visual perspective, while the remaining dots moved randomly. The proportion of dots moving congruently was parametrically varied and its specific value was described in Section 4.1.3. The avatar was located on the left or the right side of the screen, positioned 11° away from the screen center.

The auditory stimuli consisted of 500 ms white noise, which included cosine ramped rise and fall envelopes lasting for 10 ms. There were three types of sounds (“leftward”, “rightward”, and “both”) delivered through headsets. In the “leftward” or the “rightward” situations, the auditory stimulus consisted of acoustic motion, either moving from right to left (“leftward” situation) or from left to right (“rightward” situation). They were produced by decreasing the amplitude of the sound in one headset channel (from maximum to zero) while simultaneously increasing the amplitude in the other channel (from zero to maximum). Specifically, gradually decreasing loudness on the left side and increasing loudness on the right side created a “rightward” acoustic motion stimulus, while the reversed sound pattern created a “leftward” motion stimulus. In the “both” situation, auditory stimuli in both headset channels had the same amplitude, which remained constant throughout the presentation. At the beginning of the experiment, each participant completed a pure auditory block to familiarize themselves with the auditory stimuli and check their hearing. During this block, participants could adjust the maximum loudness to a comfortable level (about 60–75 dB sound pressure level).

#### 4.1.3. Design and Procedure

The independent variable of audiovisual motion direction congruency was manipulated using a within-participant design, consisting of two levels—congruent and incongruent. In the congruent condition, the moving direction of an auditory stimulus was the same as that of visual motion when perceived within the avatar-centered reference frame, while in the incongruent condition, the visual and the auditory stimuli moved in opposite directions (see Figure 2B).

Each participant’s specific 70% threshold for coherence levels of the dot motion was measured using a 2-up-1-down staircase method before they started the experimental task. This measurement was repeated four times for each participant, and the average of the four measured threshold values was used as the final threshold value. The position of the avatar was fixed during each measurement, and was ABBA counterbalanced between measurements. The procedure for these measurements was similar to that described below for the formal blocks, except that no feedback or auditory stimulus was presented during the threshold measurement. Coherence values used in the formal experiment were set at three log increments (0.85, 1, 1.33) times the measured final threshold value (16.9 ± 8.0% [*M* ± *SD*] across participants). This allowed for 6 data points (3 data points for the “leftward” and the “rightward” situations, respectively) to fit the psychophysical curve for each participant.

Each formal trial began with a fixation cross at the center of the screen, with an avatar located on the left or the right side of the fixation (see Figure 2A). After a random duration of 500–1000 ms, a group of moving dots was presented at the center of the screen, along with an auditory motion stimulus simultaneously presented in the headset. Both stimuli were presented for 500 ms. Participants were instructed to adopt the avatar’s visual perspective to judge the direction of the visual moving dots by pressing the “z” or “x” key (indicating leftward or rightward, respectively). To prevent participants from ignoring the auditory information, we also include catch trials where the auditory stimulus was in the “both” situation. Participants had to press the digital key “0” and refrain from responding to the visual target in the presence of the “both” auditory stimuli.

Each participant completed 15 blocks of trials. Each block consisted of 50 trials, including 48 formal trials and 2 catch trials, leading to a total of 30 catch trials and 720 formal trials (60 trials for each data point, in the congruent and the incongruent condition respectively). The position of the avatar, the coherence levels, the direction of the auditory motion, and the direction of the visual dots motion were randomized within each block. Participants additionally underwent a practice block of 72 trials (including 8 catch trials) at the beginning of the experiment. They received feedback at the end of each trial in the practice block, but not in the formal blocks.

### 4.2. Results

We applied the Weibull function and a nonlinear least square method [51] to fit each participant’s frequencies of “rightward” responses corresponding to each coherence level, for the congruent and the incongruent conditions, respectively (see Figure 2C). Since there were two motion directions (leftward and rightward) and three coherence levels (1, 2, and 3 representing 0.85, 1, and 1.33 log increments times each participant’s threshold value) for each direction, we obtained a total of 6 data points in each condition. We fitted these data with the Weibull function and received the optimal parameters matching these data points. Then, we figured out the slope of the fitting curve for the subsequent analysis.

A paired sample *t*-test was performed between the two levels of audiovisual motion direction congruency (congruent vs. incongruent) on the slope of the curve. Overall, motion direction congruency significantly influenced the slope of the curve, *t*(19) = 3.14, *p* = 0.005, Cohen’s *d* = 0.70. Participants were more sensitive to the change in coherence levels when the direction of the auditory motion was congruent with that of the visual motion (*M* = 1.3, *SE* = 0.1), compared to when their directions were incongruent (*M* = 1.1, *SE* = 0.1; see Figure 2D).

### 4.3. Discussion

Through investigating the influence of concurrently presented auditory motion on visual motion direction discrimination, we consistently reached the same conclusion as in Experiment 1a within dynamic perception scenarios. When the directions of visual and auditory motion were congruent in the altercentric reference frame, participants exhibited increased sensitivities to changes in the coherence levels of the moving dots, compared to the incongruent condition. These findings provide empirical evidence that when individuals adopt another person’s perspective to perceive moving visual targets, simultaneously presented non-visual (e.g., auditory) motion could also undergo altercentric processing and interact with the visual targets based on the altercentric reference frame.

Therefore, Experiments 1a and 2 investigated the existence of altercentric cross-modal interactions in explicit perspective-taking scenarios involving static and dynamic stimuli perception, respectively. However, rather than being explicitly instructed to adopt others’ perspectives, VPT typically occurs spontaneously and implicitly in daily life without explicit guidance [52,53,54]. Hence, it is crucial to explore whether such altercentric cross-modal interactions also exist during implicit VPT scenarios. Addressing this question would provide valuable insights into the adaptability and generalizability of altercentric multisensory processing.

## 5. Experiment 3a

In Experiment 3a, our objective was to investigate the persistence of altercentric cross-modal interactions in the absence of explicit instructions for perspective-taking. Specifically, we modified the experimental paradigm by introducing two visual motion stimuli and requiring participants to judge which one contained coherent moving dots. If participants could implicitly engage in perspective-taking and process visual and auditory information in an embodied manner, they might demonstrate enhanced accuracies when the directions of visual and auditory motion stimuli were congruent within the altercentric reference frame.

### 5.1. Method

#### 5.1.1. Participants

The sample size of Experiment 3a was determined through a priori analysis using G*Power 3.1 [43]. Building upon prior studies investigating audiovisual interactions, with effect sizes (Cohen’s *d*) ranging from 0.81 to 3.30 for audiovisual congruency [17,49,50], as well as considering the relatively attenuated effect of implicit processing, we empirically set a Cohen’s *d* of 0.70. With this effect size, a power of 0.90, and an alpha level of 0.05, the power analysis conducted with a paired sample *t*-test yielded an estimated sample size of 24.

Twenty-six participants from Peking University were recruited for Experiment 3a. All participants reported normal or corrected-to-normal vision and underwent hearing checks to confirm their auditory acuity within the normal range. After excluding one participant with a low hit rate in catch trials (<80%), the remaining 25 participants (17 females, *M* = 21.0, *SD* = 2.5) were included in the data analyses, with their average hit rates for visual and auditory catch trials being 88.6% ± 5.4% and 97.3% ± 4.8% (*M* ± *SD*), respectively.

#### 5.1.2. Apparatus and Stimuli

The apparatus and the stimuli employed in Experiment 3a were identical to those utilized in Experiment 2, with the exception of introducing an additional type of visual motion stimuli consisting solely of random moving dots. That is, the proportion of dots moving congruently within the newly introduced stimuli was zero.

#### 5.1.3. Design and Procedure

We employed a one-factor (audiovisual motion direction congruency: congruent vs. incongruent) within-participant design in Experiment 3a. In each trial, two visual motion stimuli were sequentially presented, with one comprising a combination of coherent and random moving dots (coherent visual motion). The coherent dots moved either upward or downward, similar to that used in Experiment 2. Conversely, the other stimulus solely consisted of random moving dots (random visual motion). Both visual motion stimuli were accompanied by the same auditory motion stimuli. In the congruent condition, the auditory stimuli moved in the same direction as the coherent visual motion when perceived from the avatar-centered reference frame. Conversely, in the incongruent condition, the auditory and the coherent visual motion stimuli moved in opposite directions within the reference frame.

Given that participants’ accuracies in distinguishing the sequence of the two visual motion stimuli were positively associated with the coherence levels, we determined each participant’s specific 70% threshold for detecting coherent visual motion using a 2-up-1-down staircase method prior to the start of the experimental task. This measurement was repeated four times per participant, and the average of these measurements was considered as their final threshold value. The procedure for these measurements closely resembled that used in the formal experiment, except that only visual motion stimuli (no avatar, auditory stimuli, or feedback) were presented during the threshold assessment. To achieve the desired auditory facilitating effect on visual motion discrimination [50,55], we maintained coherence at intermediate levels (corresponding to around the 70% discrimination threshold) throughout the experiment by dynamically adjusting its value after every one-third block. Specifically, the coherence value was divided or multiplied by 10^0.05^ based on whether the average accuracy across trials in the one-third block was above or below 70%. The new value was then utilized in the subsequent one-third block. Across all participants, the average threshold value throughout the experiment was 10.4 ± 3.7% (*M* ± *SD*). This approach aimed to prevent performance fluctuations over time and maintain consistent overall performance.

**Figure 3:**
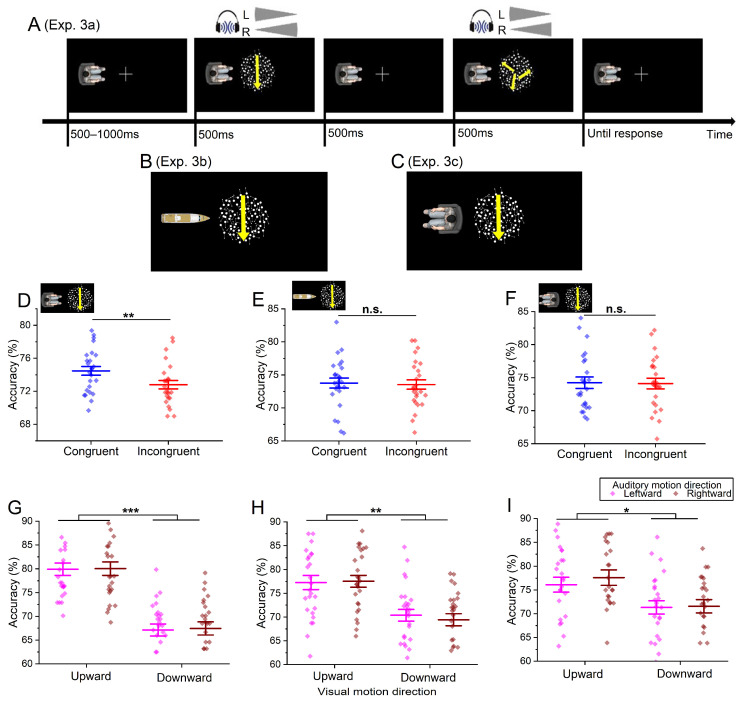
(**A**) Experimental procedure. An exemplary trial in the congruent condition in Experiment 3a, where a “rightward” auditory motion and a “rightward” coherent visual motion are presented. The directions of the coherent moving dots and the random moving dots are indicated by the yellow arrows, which are not presented during the experiment. The loudness changes in the left (L) and right (R) channels of the headset are depicted by the grey fan-shaped symbols. (**B**,**C**) Visual scenes in Experiments 3b and 3c. (**D**–**I**) Experimental results. Participants’ accuracies in the congruent and incongruent conditions in Experiments 3a (**D**), 3b (**E**), and 3c (**F**). Their accuracies in different visual (upward vs. downward) and auditory (leftward vs. rightward) motion directions in Experiments 3a (**G**), 3b (**H**), and 3c (**I**). Error bars indicate one standard error. * *p* < 0.05. ** *p* < 0.01. *** *p* < 0.001. “n.s.” indicates the lack of statistical significance.

The formal trial procedure closely resembled that of Experiment 2, with the exception that two pairs of audiovisual motion stimuli were presented sequentially (see Figure 3A). The duration of each pair of stimuli and the interval between them was 500 ms. Participants were instructed to distinguish whether the coherent visual motion appeared first or second in the sequence by pressing either the “z” or “x” key, respectively (the key arrangement was counterbalanced across participants). The purpose and the method of inserting auditory catch trials were identical to those in Experiment 2. Additionally, to rationalize the appearance of the avatar, we also included visual catch trials where no avatar was displayed. If participants noticed the absence of the avatar during a visual catch trial, they had to withhold their responses.

Each participant completed 12 blocks of trials. Each block consisted of 54 trials, including 48 formal trials and 6 catch trials (comprising 4 visual and 2 auditory), resulting in a total of 576 formal trials. The position of the avatar, the sequence of coherent visual motion and random visual motion, the direction of the visual motion, as well as the direction of the auditory motion were randomized within each block. Participants additionally underwent a practice block that was identical to one formal block at the onset of the experiment, except that feedback was provided after each trial in the practice block but not during the formal blocks.

### 5.2. Results

Participants’ accuracies in distinguishing the sequence position of coherent visual motion were significantly higher when accompanied by a congruent auditory motion stimulus (*M* = 74.5%, *SE* = 0.5%) compared to an incongruent one (*M* = 72.8%, *SE* = 0.5%), as indicated by a paired sample *t*-test, *t*(24) = 3.00, *p* = 0.006, Cohen’s *d* = 0.60 (see Figure 3D).

Subsequently, a 2 × 2 repeated-measure ANOVA was conducted on participants’ accuracies to examine whether they employed matching strategies in the task (e.g., associating upward and downward visual stimuli with leftward and rightward auditory stimuli, respectively). Visual motion direction (upward vs. downward) and auditory motion direction (leftward vs. rightward) were considered as within-participant variables. The main effect of visual motion direction was found to be significant, *F*(1, 24) = 28.18, *p* < 0.001, ŋ_p_^2^ = 0.54. Participants exhibited significantly higher accuracies in discriminating the sequence position of coherent visual motion in the upward condition (*M* = 79.9%, *SE* = 1.3%) compared to the downward condition (*M* = 67.3%, *SE* = 1.2%). However, no significant main effect of auditory motion direction was observed, *F*(1, 24) = 0.15, *p* = 0.699, ŋ_p_^2^ = 0.01. Importantly, the interaction between these two variables was not significant either, *F*(1, 24) = 0.03, *p* = 0.862, ŋ_p_^2^ < 0.01 (see Figure 3G).

### 5.3. Discussion

Although participants were instructed to respond to visual motion targets from their own perspectives, the presence of a task-irrelevant human avatar in the scene elicited an implicit perspective-taking. Specifically, we observed that participants exhibited enhanced accuracies in discerning coherent visual motion when its direction was congruent with auditory motion, compared to situations where such congruency did not exist. Since this motion direction congruency existed exclusively within the altercentric reference frame but not within the egocentric reference frame, these results suggest that participants spontaneously adopted the avatar-centered reference frame and processed visual and auditory stimuli in an embodied manner, thereby resulting in cross-modal interactions.

Importantly, these findings demonstrate that altercentric cross-modal interactions extend beyond instructed perspective-taking tasks in controlled laboratory scenarios, and instead represent a highly generalized process that could potentially occur in implicit social contexts. As previously mentioned, individuals are typically not explicitly instructed to adopt others’ viewpoints in their daily lives [56]; rather, perspective-taking often occurs automatically and unconsciously, resulting in rapid comprehension of others’ thoughts and intentions, and enhancement in social interactions [7,30,52,53,54]. Our study expands upon the existing research on implicit VPT by demonstrating the concurrent implicit processing of non-target auditory information with visual information and their cross-modal interactions in an altercentric reference frame in an embodied manner. These findings provide valuable insights into the multi-modal processing in social interaction situations.

Furthermore, we investigated whether participants engaged in spatial matching strategies in the egocentric reference frame. The findings revealed that participants failed to establish associations between visual and auditory information, as the interaction between visual motion direction and auditory motion direction was not significant. Interestingly, a higher accuracy was observed for upward visual motion stimuli compared to downward ones. This perceptual asymmetry may be attributed to the consistent influence of gravity from the Earth [57,58,59], which led participants to perceive downward motion as more plausible and exhibit heightened sensitivities toward the relatively unfamiliar upward stimuli.

## 6. Experiment 3b

Experiment 3b was conducted as a control experiment to eliminate potential confounding factors and validate that the outcomes observed in Experiment 3a were solely attributed to implicit altercentric embodied processing. In this experiment, we replaced the human avatar used in Experiment 3a with a boat that provided orientation information while lacking social characteristics [60,61]. Our hypothesis posited that the absence of a human avatar would eliminate the VPT process and embodied processing, resulting in the disappearance of cross-modal interactions within the altercentric reference frame.

### 6.1. Method

#### 6.1.1. Participants

As a control experiment, Experiment 3b employed the identical estimated number of participants (24 individuals) as Experiment 3a. Twenty-seven participants from Peking University were recruited for Experiment 3b. All participants reported normal or corrected-to-normal vision and underwent hearing checks to confirm their auditory acuity. After excluding one participant with a low hit rate in catch trials (<80%), the remaining 26 participants (15 females, *M* = 19.2, *SD* = 1.9) were included in the data analyses, with a hit rate of 86.3% ± 3.4% for visual catch trials and 96.2% ± 4.6% (*M* ± *SD*) for auditory catch trials, respectively.

#### 6.1.2. Apparatus and Stimuli

The apparatus and the stimuli remained consistent with Experiment 3a, with the exception of substituting the human avatar with an image of a boat (see Figure 3B).

#### 6.1.3. Design and Procedure

The design and the procedure employed in this study were identical to those utilized in Experiment 3a. Across all participants, the average threshold value utilized throughout the experiment was 12.0 ± 4.0% (*M* ± *SD*).

### 6.2. Result

The participants’ accuracies were compared using a paired sample *t*-test, with the independent variable being audiovisual motion direction congruency (congruent vs. incongruent). No significant difference in performance was observed between the congruent condition (*M* = 73.8%, *SE* = 0.7%) and the incongruent condition (*M* = 73.5%, *SE* = 0.7%), *t*(25) = 0.32, *p* = 0.751, Cohen’s *d* = 0.06 (see Figure 3E).

Then, to examine whether participants employed matching strategies in the task (e.g., associating upward and downward visual stimuli with leftward and rightward auditory stimuli, respectively), a 2 × 2 repeated-measure ANOVA was conducted on participants’ accuracies, with visual motion direction (upward vs. downward) and auditory motion direction (leftward vs. rightward) as within-participant variables. The main effect of visual motion direction was significant, *F*(1, 25) = 13.02, *p* = 0.001, ŋ_p_^2^ = 0.34. Participants exhibited significantly higher accuracies in discriminating the sequence position of coherent visual motion in the upward condition (*M* = 74.4%, *SE* = 1.3%) compared to the downward condition (*M* = 69.9%, *SE* = 1.1%). However, no significant main effect of auditory motion direction was observed, *F*(1, 25) = 0.26, *p* = 0.613, ŋ_p_^2^ = 0.01. More importantly, the interaction between these two variables was also not significant, *F*(1, 25) = 0.62, *p* = 0.437, ŋ_p_^2^ = 0.02 (see Figure 3H).

### 6.3. Discussion

The results of Experiment 3b suggest that the outcomes of Experiment 3a were a result of implicitly adopting the avatar’s perspective. Specifically, substituting the human avatar with an inanimate object resulted in the disappearance of the audiovisual motion direction congruency effect, indicating that this effect was primarily driven by the social and anthropomorphic characteristics inherent to the human avatar [7,60,61], and the corresponding altercentric cross-modal interactions emerged as a consequence of embodied processing rather than other factors. Furthermore, we also verified that visual and auditory information did not interact when individuals processed information in an egocentric reference frame due to spatial mismatch, thereby ruling out any influence from other spatial matching strategies.

## 7. Experiment 3c

Experiment 3c was also conducted as a control experiment to underscore the importance of the avatar’s orientation toward the scene in altercentric cross-modal interactions [62]. By rotating the avatar 180 degrees to face away from the stimuli, we hypothesized that altercentric cross-modal interactions would diminish as adopting the avatar’s visual perspective would not contribute to processing visual targets located outside its visual field.

### 7.1. Method

#### 7.1.1. Participants

Similar to Experiment 3b, the expected number of participants was 24 in Experiment 3c. Twenty-six participants from Peking University were recruited for Experiment 3c. All participants reported normal or corrected-to-normal vision and underwent a hearing examination to confirm their hearing acuity. After excluding one participant with a low hit rate in catch trials (<80%), the remaining 25 participants (15 females, *M* = 20.2, *SD* = 2.3) were included in the data analyses (their hit rate was 86.8% ± 4.9% for visual catch trials and 97.7% ± 3.0% for auditory catch trials [*M* ± *SD*], respectively).

#### 7.1.2. Apparatus and Stimuli

The apparatus and the stimuli closely resembled those in Experiment 3a, with the exception that the human avatar was facing away from the center of the screen.

#### 7.1.3. Design and Procedure

The design and procedure employed in this study were identical to those utilized in Experiment 3a, except for the modification of the human avatar’s orientation (see Figure 3C). Additionally, it was worth mentioning that the left and right sides of a facing-away avatar were opposite to those of a facing-to avatar. For instance, when presented with an upward visual motion stimulus, a left-facing-to avatar perceived it as moving leftward, while, for a facing-away avatar, it would be coded as moving rightward (relative to the orientation of the avatar’s body). A trial where auditory stimuli moved in the same direction as coherent visual motion when perceived from the avatar-centered reference frame was considered congruent. Otherwise, it was incongruent. Across all participants, the average threshold value utilized throughout the experiment was 12.0 ± 4.4% (*M* ± *SD*).

### 7.2. Result

The participants’ accuracies were compared using a paired sample *t*-test, with the independent variable being audiovisual motion direction congruency (congruent vs. incongruent). No significant difference in performance was observed between the congruent condition (*M* = 74.1%, *SE* = 0.8%) and the incongruent condition (*M* = 74.2%, *SE* = 0.9%), *t*(24) = 0.17, *p* = 0.867, Cohen’s *d* = 0.03 (see Figure 3F).

Then, to examine whether participants employed matching strategies in the task (e.g., associating upward and downward visual stimuli with leftward and rightward auditory stimuli, respectively), a 2 × 2 repeated-measure ANOVA was conducted on participants’ accuracies, with visual motion direction (upward vs. downward) and auditory motion direction (leftward vs. rightward) as within-participant variables. The main effect of visual motion direction was significant, *F*(1, 24) = 5.07, *p* = 0.034, ŋ_p_^2^ = 0.17. Participants exhibited significantly higher accuracies in discriminating the sequence position of coherent visual motion in the upward condition (*M* = 76.9%, *SE* = 1.5%) compared to the downward condition (*M* = 71.5%, *SE* = 1.3%). However, no significant main effect of auditory motion direction was observed, *F*(1, 24) = 1.65, *p* = 0.211, ŋ_p_^2^ = 0.06. Once again, the interaction between these two variables was not found to be significant, *F*(1, 24) = 0.65, *p* = 0.428, ŋ_p_^2^ = 0.03 (see Figure 3I).

### 7.3. Discussion

The results of Experiment 3c further supported the role of embodied processing in altercentric cross-modal interactions. When the avatar was oriented away from the screen’s center, the visual targets fell beyond its visual field [62]. Consequently, adopting the avatar’s visual perspective ceased to contribute to processing visual targets, therefore resulting in the disappearance of the audiovisual motion direction congruency effect within the avatar-centered reference frame. Additionally, no evidence was found for the utilization of spatial matching strategies.

## 8. General Discussion

The interaction of sensory information from diverse modalities is a fundamental process in self-perspective processing, facilitating individuals’ access to an accurate perception of the world [9]. However, when individuals adopt others’ visual perspectives to infer their visual experiences during social interactions, inputs from non-visual modalities are also received. Consequently, it raises the question of whether these multisensory inputs undergo altercentric cross-modal interactions similar to those observed in self-perspective processing. Our study aimed to address this question using a novel VPT paradigm, wherein we introduced an auditory stimulus to participants while they performed judgments on the location or the direction of a visual target from an avatar’s perspective. Specifically, participants exhibited improved performance when the auditory stimulus cued the location of the subsequent visual target from the avatar’s perspective in Experiment 1a, and an alternative explanation of auditory bias was ruled out in Experiment 1b. In Experiment 2 and 3a, we observed persistent altercentric audiovisual interactions in dynamic audiovisual scenes, wherein congruent-direction auditory stimuli significantly enhanced the participants’ performance in discriminating the direction of moving dots (Exp. 2) and distinguishing the sequence position of coherent visual motion (Exp. 3a). Furthermore, our findings showed no cross-modal interactions in Experiment 3b where no human avatar was presented and in Experiment 3c where an avatar facing away from the stimuli was presented, further substantiating the crucial role of embodied processing.

Crucially, we believe that the processes of the mental-body transformation and subsequent embodied processing play critical roles in facilitating altercentric cross-modal interactions. Previous studies have demonstrated that immersive encoding of visual stimuli from the adopted visual perspective, achieved through embodied processing, enables individuals to acquire a vivid representation of another person’s visual experience [5,6,7]. As for the auditory information, since embodied processing contains people’s imagination of standing in the adopted person’s location [5], the auditory stimuli received through the headset would seem to be received from the left or the right side of the avatar’s head (the location of the “virtual self” after the mental-body transformation). Taken together, through mentally transforming to others’ locations and engaging in embodied processing, people unified visual and auditory information to the altercentric reference frame and integrated the spatial-consistent information in an immersive manner, thus permitting the altercentric interactions of sensory information from different modalities.

Our study has also advanced the current understanding of cross-modal interactions by revealing that the interplay between multisensory information exists beyond the egocentric reference frame that people usually adopt. Particularly, the transformation-uniform theory proposed in the egocentric integration field provided us with an idea to uncover the mechanisms of the altercentric cross-modal interactions [32]. This theory proposes that the brain performs audiovisual integration by creating a unified spatial representation of visual and auditory stimuli by converting them into a common reference frame [32,37]. Then, this unification process might also apply to the altercentric cross-modal interactions when people adopt another person’s visual perspective. But compared with the egocentric audiovisual interactions where information was converted within the egocentric reference frame (e.g., auditory information was transformed from the head-centered to the eye-centered reference frame [36,37]), the altercentric audiovisual interactions were based on a transformation out of the egocentric reference frame (from the egocentric to an altercentric reference frame [4]). That is, when people transformed their bodies in imagination to another person’s location during VPT, the sensory information they received from multiple modalities would be unified to the altercentric reference frame, allowing people to assess whether they were corresponding in space.

Meanwhile, our findings also revealed that not only those instructed stimuli but also the non-target stimuli could be processed in an embodied manner during a VPT task. However, another study found somewhat inconsistent evidence regarding the processing of non-target stimuli during VPT. Specifically, in Samuel et al.’s study [63], individuals appeared to disregard real-world obstacles when engaging in the mental-body transformation and exhibited a reckless approach to completing the VPT task. Consequently, they were inclined to regard embodied processing as a limited process where only the self and the target object, but not features of the environment would be treated. We posit that the modality specificity of attentional resources may contribute to the disparity observed between Samuel et al.’s findings and ours [64,65,66]. It is plausible that cognitive resources are more likely to be limited and competitive within one single modality, rather than across different modalities. Therefore, due to resource limitations, task-irrelevant stimuli within the same sensory modality as the targets might be disregarded and excluded from attention, while those from a different modality might have sufficient resources for processing. However, this hypothesis requires future investigations.

Furthermore, it is noteworthy that individuals could engage in altercentric cross-modal interactions even in spontaneous and implicit perspective-taking scenarios, extending beyond instructed situations. In everyday social interactions, individuals rarely receive explicit instructions to adopt others’ perspectives to perceive the world; rather, they spontaneously do so during natural communications to comprehensively and promptly acquire information [56]. Previous studies have demonstrated that when individuals viewed an object together with someone else from different viewpoints, they automatically generated visual representations from that person’s perspective while simultaneously processing the target from their own viewpoint [52,53,67,68]. Our study extended these findings by showing that besides visual stimuli, multisensory inputs could also be implicitly processed and interact with each other from others’ perspectives. This finding enhances our understanding of the underlying mechanism through which people implicitly simulate others’ perceptual experiences in social contexts.

It is worth considering that alternative strategies might have been employed by participants in VPT tasks, as suggested by previous researchers [23,69,70,71,72,73]. For example, participants might utilize a mental-object rotation (mentally rotating the visual stimuli from the avatar’s viewpoint to their own perspectives) or a perspective calculation (where people make spatial inferences between the self- and other-perspective, such as “if he is facing me, then my right will be his left”) strategy to complete the VPT task. However, both strategies are usually utilized in explicit VPT tasks. That is, participants had no reason to spontaneously rotate an object or calculate the spatial relationships between objects from an altercentric perspective (a process that consumes resources) when they were processing from their own perspectives, as was the case in Experiment 3a. Consequently, if participants did indeed employ these two strategies during instructed VPT tasks, the predicted cross-modal effects should not have occurred in Experiment 3a. Nevertheless, this hypothesis contradicts our finding of implicit altercentric cross-modal interactions. Additionally, the use of motion stimuli in our study effectively circumvented potential reliance on the mental-object rotation strategy, as mentally rotating a group of moving stimuli posed greater challenges compared to static ones [74]. Collectively, these pieces of evidence suggest that neither mental-object rotation nor perspective calculation was likely to be employed.

When it comes to cross-modal interactions, some researchers propose that instead of the transformation-uniform view, people could alternatively establish point-to-point spatial links between the two reference frames to assess whether information corresponded in space or not [75,76,77,78]. Nevertheless, considering the frequent variations in distance and heading angle discrepancy between the observer and the adopted person in daily life (which means the spatial relationships between the egocentric and the altercentric reference frames need to be updated frequently), performing embodied processing to unify sensory information to one reference frame was more efficient and resource-conserving compared to establishing spatial links. Moreover, cross-modal interactions can occur not only in spatial dimensions but also in time dimensions [79,80]. We propose to investigate the possibility of altercentric cross-modal interactions by manipulating congruency in temporal properties. We believe one of the challenges lies in designing information from two modalities to correspond temporally or rhythmically within an altercentric reference frame while mitigating potential interference from egocentric cross-modal interactions.

Finally, we employed an avatar as a surrogate whose visual perspective was adopted by participants in our experiments, potentially compromising the ecological validity of our study. In future investigations, it is suggested to delve deeper into understanding how social characteristics (e.g., emotional valence and trustworthiness [81,82]) of a real person whose perspective is adopted, as well as the relationship between participants and that person (e.g., social relations, interpersonal attitudes, and affiliation congruities [83,84]) influence altercentric cross-modal interactions. Additionally, future studies can investigate whether the effects observed in this study could be extended to different demographic groups. It is well-established that individuals belonging to different demographic groups exhibit dissimilar propensities in adopting others’ perspectives. For instance, older individuals exhibit a reduced tendency to consider information from others’ perspectives compared to younger individuals [85]. Meanwhile, individuals diagnosed with autism spectrum disorder often exhibit limited abilities in perspective-taking when compared to typically developing individuals [86,87]. Therefore, subsequent research should include more diverse samples to enhance the generalizability of our findings and explore the role of the perspective-taking ability in the altercentric cross-modal interactions. Furthermore, considering information from proximal sense modalities (e.g., tactile and taste [88]) may be more readily processed in an embodied manner, it is imperative to expand investigations on altercentric audiovisual interactions to encompass additional modalities, such as visual-tactile interactions. By doing so, we can further elucidate the distinctive characteristics inherent in these types of interactions. Lastly, in addition to previous studies demonstrating the enhancement of cross-modal interactions in individuals’ perception of their physical environment [10,11,12], our findings further extend this phenomenon to social interactions. Therefore, future research could capitalize on the benefits of multisensory processing to augment perspective-taking abilities among individuals with limited skills in understanding others. For instance, incorporating auditory cues for astronauts with low perspective-taking abilities may assist them in adopting camera-centered perspectives when manipulating robotic arms. Moreover, it is suggested to consider a novel dimension of multisensory processing while assessing individuals’ perspective-taking tendencies [1,2,3].

## 9. Conclusions

In summary, the current study for the first time provides evidence that auditory information could be processed within an altercentric reference frame during the adaptation of others’ visual perspectives and further demonstrates that cross-modal interactions occurred in an embodied manner within this altercentric reference frame. Our findings not only expand the exploration of cross-modal interactions from egocentric to altercentric reference frames but also offer valuable insights into the embodied processing theory by elucidating low-level multisensory processing and revealing automatic interactions of multisensory information during VPT.

## Data Availability

Raw data and analysis code are publicly available at https://osf.io/29t36/ (accessed on 23 March 2024).
